# Mitochondrial calcium cycling in neuronal function and neurodegeneration

**DOI:** 10.3389/fcell.2023.1094356

**Published:** 2023-01-24

**Authors:** Grant C. Walters, Yuriy M. Usachev

**Affiliations:** Department of Neuroscience and Pharmacology, Iowa Neuroscience Institute, University of Iowa, Iowa City, IA, United States

**Keywords:** mitochondria, calcium, MCU, neurodegeneration, neuronal calcium homeostasis

## Abstract

Mitochondria are essential for proper cellular function through their critical roles in ATP synthesis, reactive oxygen species production, calcium (Ca^2+^) buffering, and apoptotic signaling. In neurons, Ca^2+^ buffering is particularly important as it helps to shape Ca^2+^ signals and to regulate numerous Ca^2+^-dependent functions including neuronal excitability, synaptic transmission, gene expression, and neuronal toxicity. Over the past decade, identification of the mitochondrial Ca^2+^ uniporter (MCU) and other molecular components of mitochondrial Ca^2+^ transport has provided insight into the roles that mitochondrial Ca^2+^ regulation plays in neuronal function in health and disease. In this review, we discuss the many roles of mitochondrial Ca^2+^ uptake and release mechanisms in normal neuronal function and highlight new insights into the Ca^2+^-dependent mechanisms that drive mitochondrial dysfunction in neurologic diseases including epilepsy, Alzheimer’s disease, Parkinson’s disease, and amyotrophic lateral sclerosis. We also consider how targeting Ca^2+^ uptake and release mechanisms could facilitate the development of novel therapeutic strategies for neurological diseases.

## Introduction

Mitochondria are colloquially referred to as the powerhouse of the cell because of their critical role in the production of ATP through oxidative phosphorylation (OXPHOS) (Brookes et al., 2004; Nunnari and Suomalainen, 2012). The production of ATP through OXPHOS is especially important within the brain, which accounts for nearly 20% of the body’s resting metabolism (Attwell and Laughlin, 2001; Howarth et al., 2012). Due to the high energy demand of neurons, they are very susceptible to mtDNA mutations that disrupt the electron transport chain; these mutations can result in a variety of neurological dysfunctions including seizures, blindness, and intellectual disabilities (DiMauro and Schon, 2003). In addition to producing ATP, reactive oxygen species (ROS) are produced as a byproduct of OXPHOS.

Mitochondrial ROS production is associated with damage to lipids, proteins, and nucleic acids, and excessive ROS are implicated in neurodegeneration (Nunnari and Suomalainen, 2012; Beckhauser et al., 2016). Super oxide free radicals (O_2_^−^) produced through OXPHOS are converted to H_2_O_2_ by enzymes such as superoxide dismutase and subsequently broken down into harmless byproducts (Beckhauser et al., 2016; Angelova and Abramov, 2018). When ROS are not broken down, the resulting oxidative stress can lead to activation of apoptotic signaling cascades and thereby to cell death (Fleury et al., 2002; Ueda et al., 2002). However, ROS also play an important physiological role, regulating signaling pathways that are critical for a wide variety of cellular functions. In neurons, ROS signaling is critical for axon development (Beckhauser et al., 2016), it modulates expression of AMPA and NMDA glutamate receptors (Esteras et al., 2022), and regulates various forms of synaptic plasticity including long-term potentiation (LTP) (Klann, 1998; Beckhauser et al., 2016). Despite the importance of ROS for normal neuronal function, proper regulation of their production is critical, because overproduction and subsequent oxidative stress are implicated in multiple pathologies. These include Alzheimer’s disease, Parkinson’s disease, Huntington’s disease, Epilepsy, and schizophrenia (Bezprozvanny, 2009; Liochev, 2013; Puttachary et al., 2015; Beckhauser et al., 2016; Angelova and Abramov, 2018; Dagda, 2018).

**FIGURE 1:**
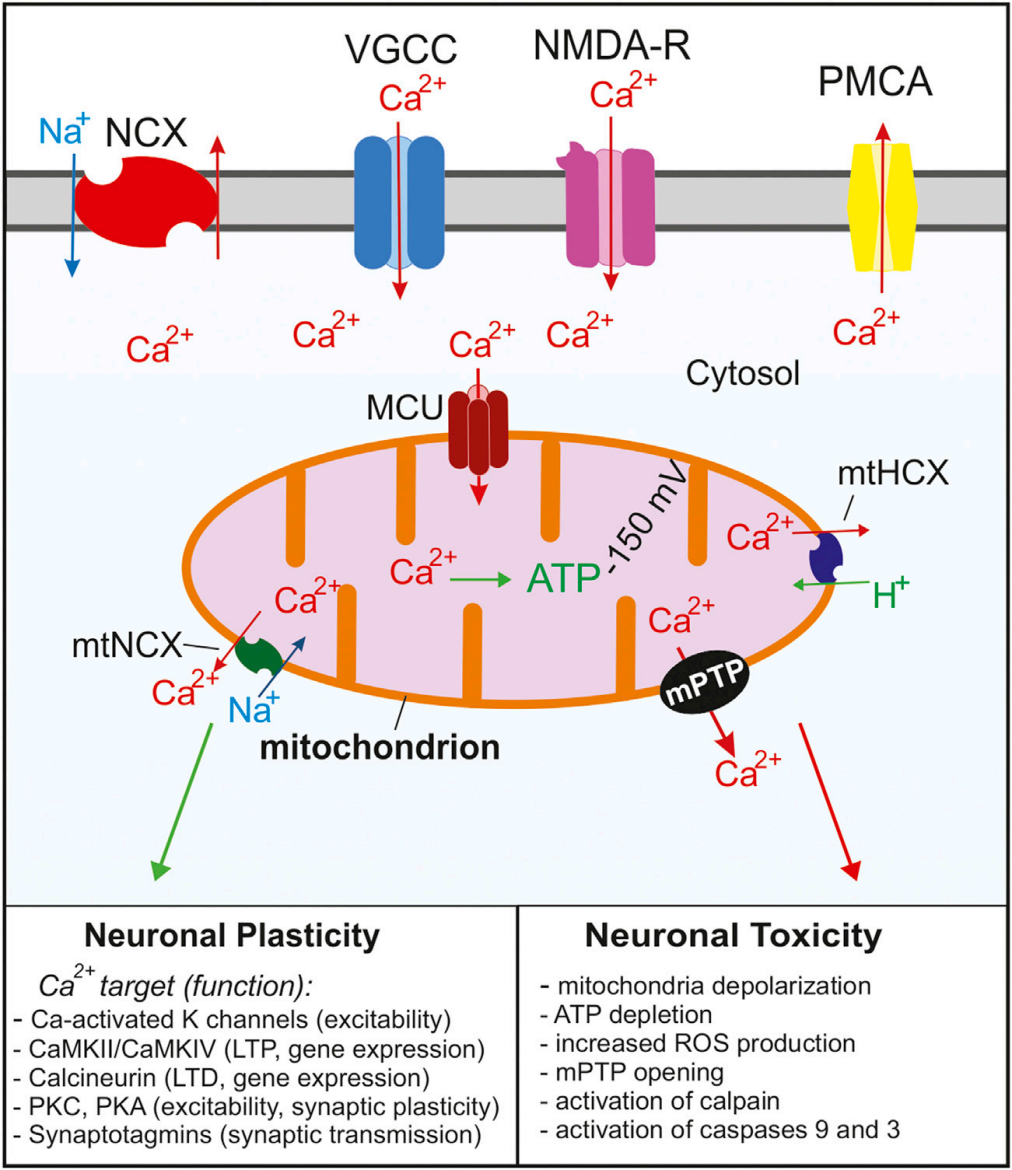
Summary of the Ca^2+^ signaling mechanisms in neurons that regulate cytosolic and mitochondrial Ca^2+^ concentrations. Ca^2+^ enters the cell through voltage gated Ca^2+^ channels (VGCC) following depolarization or through glutamate NMDA receptors. As the cytosolic Ca^2+^ concentration increases, the MCU complex opens and Ca^2+^ enters the mitochondria. Ca^2+^ is rapidly removed from the cytosol *via* Na^+^/Ca^2+^ exchangers (NCX) and plasma membrane Ca^2+^ ATPases (PMCA). As the cytosolic Ca^2+^ concentration decreases Ca^2+^ is slowly released from the mitochondria through Na^+^/Ca^2+^ (mtNCX) exchangers and H^+^/Ca^2+^ exchangers (mtHCX). Proper Ca^2+^ buffering is critical for normal neuronal functions and neuronal plasticity. Impaired buffering and Ca^2+^ overload leads to the opening of the mPTP and activation of apoptotic pathways and is implicated in many neurological diseases. For simplicity, the ER and other Ca^2+^ organelles are omitted from the model.

In addition to ATP synthesis and ROS generation, mitochondria also play a critical role in Ca^2+^ signaling. Mitochondria efficiently buffer Ca^2+^ entering the cell during neuronal excitation and then slowly release accumulated Ca^2+^ back to the cytosol; this limits the amplitude and prolongs the duration of cytosolic Ca^2+^ responses. Consequently, mitochondrial have a tremendous ability to control and modulate many Ca^2+^-dependent functions in neurons, including neuronal excitability, neurotransmitter release, synaptic plasticity, gene expression, and cell survival (Mattson et al., 2008; Pivovarova and Andrews, 2010; Brini et al., 2014; Devine and Kittler, 2018; Jung et al., 2020) (Figure 1). Ca^2+^ uptake into the mitochondrial matrix regulates ATP synthesis by activating multiple components of the OXPHOS pathway, including ATP synthase, α-ketoglutarate dehydrogenase, isocitrate dehydrogenase, and pyruvate dehydrogenase (Denton et al., 1972; Brookes et al., 2004; Denton, 2009; Tarasov et al., 2012). ATP production depends on the concentration of Ca^2+^ within the mitochondrial matrix, with submicromolar mitochondrial Ca^2+^ concentrations stimulating ATP synthesis, and mitochondrial Ca^2+^ concentrations above 10 μM suppressing ATP production (Fink et al., 2017). While the Ca^2+^ dependent production of ATP is conserved across most cells within the body, neurons have unique functions that are dependent upon Ca^2+^ signaling as discussed below.

## Mitochondrial Ca^2+^ uptake and release mechanisms

### General characteristics

In neurons at rest, total calcium concentration in the matrix is very low, usually <100 nM (Pivovarova et al., 1999; Stanika et al., 2012). During electrical or synaptic stimulation, total mitochondrial calcium concentration can increase to millimolar levels within seconds (Pivovarova et al., 1999; Pivovarova et al., 2002). In the case of modest to strong stimulation, the rate of mitochondrial Ca^2+^ uptake in intact neurons can be as high as 200 μM/s relative to the mitochondrial volume (Pivovarova et al., 1999; Colegrove et al., 2000a). Ca^2+^ transport from the cytosol into the mitochondrial matrix requires crossing the outer and inner mitochondrial membranes (OMM and IMM, respectively). The OMM is permeable to molecules smaller than 5 kDa, including Ca^2+^. This permeability is ascribed to voltage-dependent anion channels (VDACs) also known as mitochondrial porins that are highly expressed in the OMM (Spat et al., 2008; Shoshan-Barmatz et al., 2010; Rizzuto et al., 2012). It is generally assumed that Ca^2+^ concentration in the intermembrane space is the same as in the cytosol. Ca^2+^ transport across the IMM is mediated by a designated transporter, mitochondrial Ca^2+^ uniporter, and is driven by a steep electrical potential across the IMM (ΔΨ_mt_ ∼ −150 mV) (Friel, 2000; Thayer et al., 2002; Nicholls, 2005). Recent electrophysiological and molecular studies strongly suggest that the mitochondrial Ca^2+^ uniporter functions as a Ca^2+^-selective ion channel, which is consistent with the very high rate of Ca^2+^ uptake by mitochondria (Kirichok et al., 2004; De Stefani et al., 2011; Fieni et al., 2012; Chaudhuri et al., 2013). The mitochondrial Ca^2+^ uniporter channel is gated by Ca^2+^, and the rate of mitochondrial Ca^2+^ uptake depends on the cytosolic Ca^2+^ concentration [Ca^2+^]_i_ as a power function of ∼2–2.5 (Gunter and Pfeiffer, 1990; Colegrove et al., 2000a; Nicholls, 2012; Shutov et al., 2013). Earlier studies in isolated mitochondria suggested that the EC_50_ of the [Ca^2+^]_i_ for uniporter activation is ∼10–20 μM (Gunter and Pfeiffer, 1990). Such a requirement implied that mitochondria are involved in Ca^2+^ transport only in a setting of intense stimulation or pathological [Ca^2+^]_i_ elevations caused by toxic conditions. However, most recent studies using intact neurons indicate that mitochondrial Ca^2+^ uptake can be induced by [Ca^2+^]_i_ elevations as low as 200–300 nM, and that mitochondrial Ca^2+^ transport contributes to many aspects of Ca^2+^ signaling in neurons, both under physiological and pathological conditions (Figure 1) (Werth and Thayer, 1994; Stout et al., 1998; David, 1999; Pivovarova et al., 1999; Colegrove et al., 2000a; Pivovarova et al., 2004; Medvedeva et al., 2008; Kim and Usachev, 2009; Chouhan et al., 2010; Shutov et al., 2013).

Within the matrix, free Ca^2+^ is rapidly buffered by its incorporation into Ca-phosphate complexes, primarily in the forms of Ca_3_(PO_4_)_2_ and CaHPO_4_ (Chalmers and Nicholls, 2003; Kristian et al., 2007). Heavy Ca^2+^ buffering in the matrix helps to explain why mitochondrial Ca^2+^ concentration typically does not exceed 1–5 μM in neurons even after strong stimulation (David, 1999; Billups and Forsythe, 2002; Chalmers and Nicholls, 2003; David et al., 2003). The ratio of bound:free Ca is a dynamic value that increases as the Ca load of mitochondria increases (Chalmers and Nicholls, 2003). In neurons at rest or with a low mitochondrial Ca load, the ratio is estimated to be ∼4,000:1, and as mitochondrial Ca approaches toxic levels the ratio increases to as much as 150,000:1 (Babcock et al., 1997; Chalmers and Nicholls, 2003; Pivovarova et al., 2004; Stanika et al., 2012).

Ca^2+^ accumulated in mitochondria can be released *via* at least three mechanisms, Na^+^/Ca^2+^ exchanger, H^+^/Ca^2+^ exchanger, and the mitochondrial permeability transition pore (mPTP; Figure 1) (Bernardi, 1999; Thayer et al., 2002; Nicholls, 2005; Pivovarova and Andrews, 2010; Jung et al., 2020). It is generally agreed that under physiological conditions the mitochondrial Na^+^/Ca^2+^ exchanger (mtNCX) is the main pathway mediating Ca^2+^ efflux from mitochondria in neurons (Friel, 2000; Thayer et al., 2002; Nicholls, 2005; Jung et al., 2020; Rysted et al., 2021; Yang et al., 2021). Because the rate of Ca^2+^ efflux from mitochondria is significantly slower than that of Ca^2+^ uptake, Ca^2+^ rapidly accumulates in mitochondria following its entry into the cell during stimulation. Using an approach combining pharmacological tools and mathematical modeling, Friel and colleagues estimated that Ca^2+^ is removed from neuronal mitochondria at 35–75 nM/s, a rate ∼1000-fold slower than the rate of mitochondrial Ca^2+^ uptake (Colegrove et al., 2000a; b). However, EPMA-based examination of sympathetic neurons showed that total matrix Ca^2+^ recovered from depolarization-induced peak value of 22 mM–0.9 mM within 15 min, which yields a much faster rate value of ∼23 μM/s (Pivovarova et al., 1999). Removal of 1 Ca^2+^ by mtNCX is coupled to mitochondrial uptake of 3 Na^+^ (Bernardi, 1999; Palty and Sekler, 2012). Thus, mtNCX is electrogenic, and its activity is predicted to be reduced by mitochondrial depolarization.

While uptake of Ca^2+^ ions into the mitochondria has been studied for many decades, the molecular mechanisms by which Ca^2+^ enters and exits the mitochondria have been discovered only over the past 10 years and are discussed below (Drago et al., 2011; Kamer and Mootha, 2015; De Stefani et al., 2016; Garbincius and Elrod, 2022).

### The mitochondrial Ca^2+^ uniporter (MCU)

**FIGURE 2:**
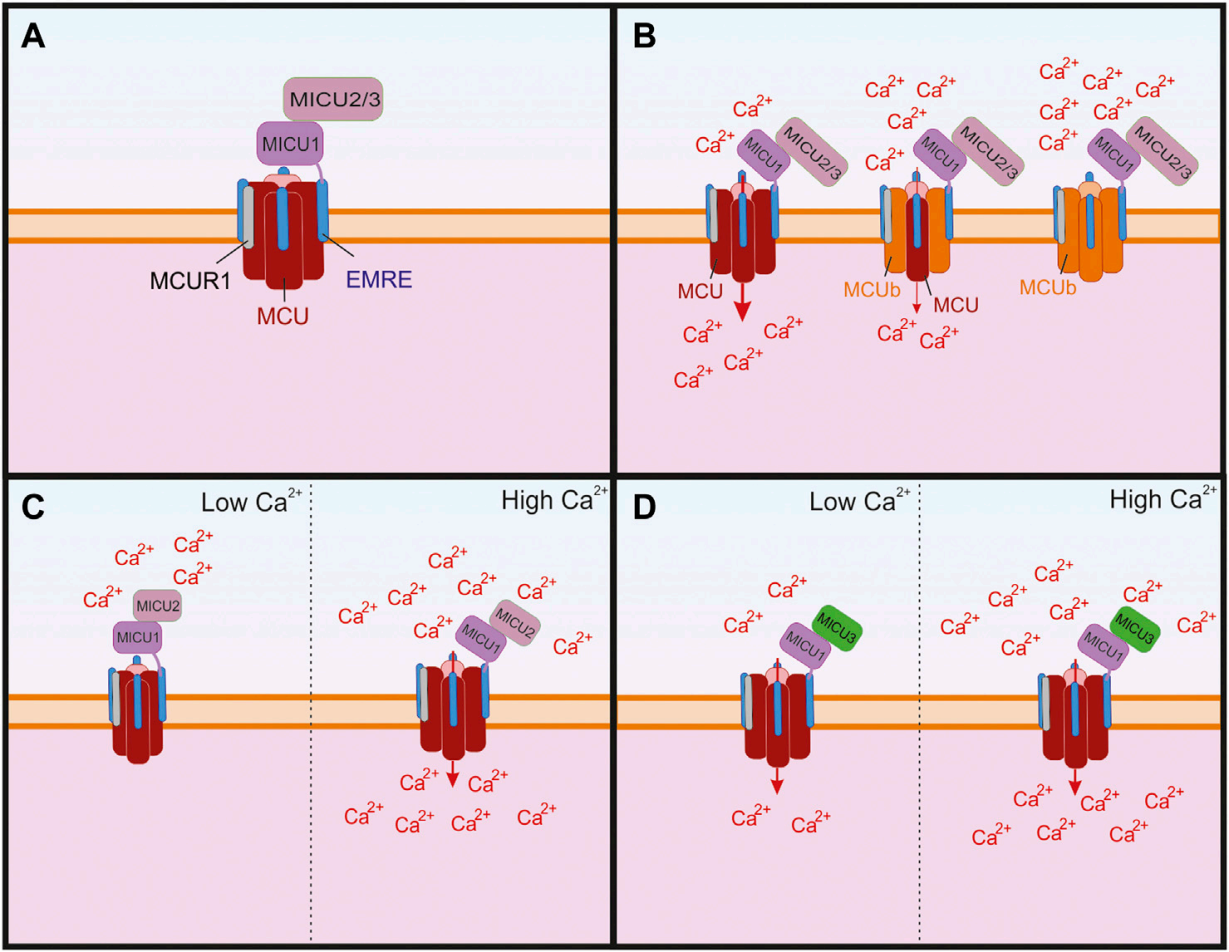
The structure of the greater mitochondrial Ca^2+^ uniporter (MCU) complex and its subunits that can regulate its permeability. **(A)** The assembled complex containing a tetramer of pore forming MCU and four subunits of the essential MCU regulator (EMRE), which are critical for the proper assembly of the transmembrane MCU to the mitochondrial Ca^2+^ uptake isoforms 1, 2, and 3 (MICU 1–3). The MCU complex also contains the MCU regulator 1 (MCUR1), which is thought to bind to EMRE. **(B)** The MCU paralog MCUb has been shown to replace subunits of pore forming MCU, which leads to reduced Ca^2+^ through the channel and as more MCUb subunits replace MCU the Ca^2+^ conductivity is reduced. **(C)** MICU1 and MICU two are critical for the Ca^2+^ sensing function of the channel and MICU2 has been shown to regulate Ca^2+^ sensitivity. When Ca^2+^ is low, MICU1 blocks the pore, but in the presence of high Ca^2+^ the conformational changes in MICU1 and MICU2 result in opening of the channel and allow Ca^2+^ to enter the mitochondria. **(D)** In neurons, MICU3 is more abundant and has been shown to replace MICU2 in dimers with MICU1. This replacement leads to increeased sensitivity to cytosolic Ca^2+^ of the MCU complex and results in MCU channel opening to lower concentrations of Ca^2+^ than compared to MICU2.

In 2004, Kirichok and Clapham demonstrated that mitochondrial Ca^2+^ uniporter functions as a Ca^2+^-selective ion channel (Kirichok et al., 2004). In 2011, two groups identified MCU (mitochondrial Ca^2+^ uniporter) as a pore-forming subunit of the greater uniporter complex (Baughman et al., 2011; De Stefani et al., 2011), and two years later, a MCU paralog and negative regulator of the complex, MCUb, was identified (Raffaello et al., 2013). MCU heteromerizes with MCUb to form a tetrameric pore structure within the inner mitochondrial membrane (Raffaello et al., 2013; Baradaran et al., 2018; Wang et al., 2019) (Figure 2). Several additional components essential for the function of the MCU complex were recently discovered. These include: mitochondria Ca^2+^ uptake isoforms 1, 2, and 3 (MICU1, MICU2, and MICU3), which are auxiliary subunits that regulate Ca^2+^-dependent gating of the channel; the mitochondrial Ca^2+^ uniporter regulator 1 (MCUR1), and an essential MCU regulator (EMRE), the protein critical for the assembly of MCU complex (Mallilankaraman et al., 2012a; Mallilankaraman et al., 2012b; Csordás et al., 2013; Sancak et al., 2013; Tomar et al., 2016; Patron et al., 2019; Garg et al., 2021). The functions of these subunits are discussed in greater detail later.

In addition to determining tertiary structure of the MCU pore complex, recent studies explored the ion selectivity regions that are conserved across multiple species, with the aim of identifying the selectivity filters within MCU and MCUb. These studies revealed that the cation selectivity of the MCU subunits is determined by the critical residues Asp240 (D) and Glu243 (E) within the carboxylate rings at the mouth of the channel, which form a so-called DXXE motif that is critical for Ca^2+^ selectivity (Oxenoid et al., 2016; Cao et al., 2017). Notably, whereas the substitution of Asp240 to Glu results in a partially functional channel, the substitution of Glu243 to Asp completely inhibits Ca^2+^ uptake (Oxenoid et al., 2016). Previous work also found that mutation of Asp240 or Glu243 to alanine (A) completely inhibited Ca^2+^ uptake through MCU (Baughman et al., 2011).

Defining the roles of MCU in the central and peripheral nervous systems *in vivo* remains a critical task in the field of mitochondrial biology. Although many *in vitro* studies (e.g., in cultured neurons or brain slices) suggested that mitochondrial Ca^2+^ transport is critical to neuronal function, studies in MCU knockout (KO) mice revealed that MCU deletion had surprisingly limited effects *in vivo* (Pan et al., 2013). The development of viable MCU KO mice was a surprise due to the importance of MCU for Ca^2+^ passage into the mitochondria. MCU KO mice were only viable on a CD1 background and KO offspring were not born at Mendelian ratios, but C57BL/6 mice were not viable due to embryonic lethality between E11.5-E13.5 (Pan et al., 2013; Murphy et al., 2014; Murphy and Steenbergen, 2021). The exact cause of embryonic lethality is not known, but it is hypothesized to be due to myocardial development issues (Murphy et al., 2014). Mitochondria from MCU KO mice did not uptake Ca^2+^, but it was not protective from cardiac ischemic reperfusion injuries (Pan et al., 2013; Murphy and Steenbergen, 2021). However, deletion of MCU from adult mice using a tamoxifen inducible model results in protection from cardiac ischemic reperfusion injuries (Kwong et al., 2015; Luongo et al., 2015). Mitochondria from global MCU KO mice have been shown to have MCU independent Ca^2+^ uptake, which could explain its therapeutic limitations in the ischemic reperfusion model (Hamilton et al., 2018). MCU independent Ca^2+^ uptake is blocked by the MCU inhibitor Ru360, and therefore it is hypothesized that the other pore forming component MCUb may be capable of compensating for the loss of MCU (Hamilton et al., 2018). MCU independent uptake could also possibly occur through mtNCX functioning in reverse mode and Letm1 (Palty et al., 2010; Jiang et al., 2013). Interestingly, MCU KO mice did not show any deficit in motor function or cognitive performance (Szibor et al., 2020), suggesting that MCU is not critical for baseline neuronal function, but plays a more important role during states of high neuronal activity, stress, neuronal injury, or in disease states. Indeed, a recent study using a mouse high-fat diet model of peripheral diabetic neuropathy showed that the deletion of MCU from peripheral neurons prevented axonal degeneration. Specifically, the deletion of MCU prevented the accumulation of Ca^2+^ in the mitochondria, subsequent Ca^2+^ induced mitochondrial disruption, and eventual loss of nociceptive fibers (George et al., 2021). Inducible neuron-specific MCU KO also protected neurons from hypoxic-ischemic injury (Nichols et al., 2018), in contrast to findings in mice with global deletion of MCU (Pan et al., 2013). The exact mechanisms by which global and inducible MCU KO differ are not known, but compensatory mechanisms, which were discussed above, may occur over time and lead to the differences observed in these studies. In another study, deletion of MCU from mice carrying the Cdh23^ahl^ allele, which causes hearing loss, led to earlier onset of high frequency hearing loss and subsequent significant hearing loss when compared to controls. By 3 months of age, there was also a loss of close to 18% of outer hair cells, but the development of the cochlea was normal (Manikandan et al., 2021). Collectively, these studies suggest that MCU either promote or reduce neuronal survival, with the outcome depending on the developmental state, cellular context, and disease conditions.

### Mitochondrial Ca^2+^ uniporter paralog (MCUb)

The pore forming subunit of MCU is surrounded by multiple proteins that make up the greater MCU complex (Figure 2). One of these is MCUb, another pore-forming subunit sharing many structural similarities with MCU (Raffaello et al., 2013). Like MCU, it contains two transmembrane domains. Its protein sequence shares 50% similarity with that of MCU, and it can form hetero-oligomers with MCU. A substitution within the DXXE motif of MCUb is hypothesized to affect its Ca^2+^ permeability. Therefore, MCUb has been proposed to function as a dominant negative regulator of the MCU complex (Raffaello et al., 2013). A recent study revealed a potential new structural role for MCUb in the greater MCU complex (Lambert et al., 2019). Specifically, it showed that the presence of MCUb disrupts the tertiary structure of the complex by competitively displacing MCU from the greater complex and reducing the association of MCU with regulatory proteins MICU1/2. This coincided with reduced function of the complex and disrupted Ca^2+^ uptake into the mitochondria (Lambert et al., 2019; Garbincius and Elrod, 2022). A recent *in vivo* study provided additional support for MCUb as a negative regulator of Ca^2+^ uptake into mitochondria (Huo et al., 2020). In this study, the deletion of MCUb in mice led to increased susceptibility to damage from ischemic-reperfusion injury in the heart, whereas overexpression of MCUb conferred some protection from cardiac remodeling and damage from the infarct (Huo et al., 2020). Studies also revealed that MCUb expression increases following cardiac injury. This led to the hypothesis that the upregulation of MCUb has evolved to prevent mitochondrial Ca^2+^ overload in response to ischemic damage (Lambert et al., 2019; Huo et al., 2020). In contrast to these findings, MCUb is upregulated in a type 2 diabetic mouse heart, and the introduction of a dominant-negative MCUb transgene into the type 2 diabetes mouse model reversed the cardiac defects, as well as the metabolic aspect of the phenotype suggesting detrimental effects of MCUb expression in this model (Cividini et al., 2021). These results are somewhat contrary to the suggestions in previous MCUb studies that the overexpression of MCUb is cytoprotective. It could be that the overexpression of MCUb is initially protective, but that chronic overexpression results in deficits in energy production leading to negative outcomes.

MCUb is also found in brain mitochondria (Hamilton et al., 2018), although its role in neurons remains unknown. Given the protective function of MCUb in the heart (Lambert et al., 2019; Huo et al., 2020) and the detrimental role of mitochondrial Ca^2+^ overload in neurons (Mattson et al., 2008; Briston et al., 2019; Jung et al., 2020; Modesti et al., 2021), it is logical to predict that MCUb plays neuroprotective roles in stroke and neurodegenerative diseases. Future work will determine if this is the case.

### MICU1-3

One of the regulatory subunits of the MCU complex was described before MCU was discovered and is encoded by the gene *CBARA1,* which was renamed to mitochondrial Ca^2+^ uptake 1 (MICU1). It is ubiquitously expressed throughout the body including the brain (Plovanich et al., 2013; Patron et al., 2019). MICU1 interacts with MCU *via* EMRE and has two EF hand domains that are critical for its Ca^2+^ sensing function (Perocchi et al., 2010; Baughman et al., 2011). Knockdown of MICU1 leads to inhibition of mitochondrial Ca^2+^ uptake in response to stimulation, yet deletion of MICU1 results in chronically elevated Ca^2+^ concentrations within the mitochondrial matrix (Mallilankaraman et al., 2012b; Csordás et al., 2013). The physiological importance of MICU1 has been demonstrated in both humans and mice. Indeed, loss-of-function mutations in MICU1 are associated with learning and motor deficits in humans (Logan et al., 2014). Similarly, neuron-specific deletion of MICU1 in mice leads to abnormal motor and cognitive phenotypes, which are likely caused by neuronal degeneration in the spinal cord and the brain (Liu et al., 2016; Singh et al., 2022). Mechanistically, the loss or deficit in MICU1 function results in chronic elevation of mitochondrial Ca^2+^ concentration and impairment of mitochondrial Ca^2+^ signaling, which leads to altered neuronal excitability, increased susceptibility to excitotoxic stress and neuronal death dependent on mitochondrial permeability transition pore (Logan et al., 2014; Singh et al., 2022). Thus, MICU1 represents a critical component of mitochondrial Ca^2+^ signaling in neurons.

MICU1 has two paralogs, MICU2 and MICU3 (Plovanich et al., 2013). MICU2 is ubiquitously expressed throughout the body, whereas MICU3 is found primarily in the nervous system (Plovanich et al., 2013; Patron et al., 2019; Ashrafi et al., 2020). Both proteins interact with MICU1 to control Ca^2+^-dependent gating of the MCU channel complex (Kamer and Mootha, 2015; De Stefani et al., 2016; Garbincius and Elrod, 2022) (Figure 2).

MICU1 and MICU2 exist as a heterodimer and have opposing regulatory effects on MCU. When the cytosolic Ca^2+^ concentration is low, MICU2 reduces MCU activity, but when the Ca^2+^ concentration is higher MICU1 increases MCU activity (Patron et al., 2014). Notably, the presence of a disulfide bond between MICU1 and MICU2 is critical for its biochemical behavior, enabling them to physically adjust their positions in response to changes in Ca^2+^ levels; when the disulfide bond is removed mitochondrial Ca^2+^ uptake increases (Petrungaro et al., 2015). A recent structural analysis of the MCU complex showed that at low Ca^2+^ concentrations MICU1 interacts closely with MCU to seal the pore, and that MICU2 is bound to MICU1 but does not directly interact with MCU (Fan et al., 2020). The same study showed that at high levels of Ca^2+^ (2 mM) the MCU complex forms a dimer with a second MCU complex and in this arrangement the MICU2 subunits interact directly. This also results in MICU1 moving away from the pore of the MCU complex, allowing for Ca^2+^ uptake (Fan et al., 2020). In addition to playing a critical role in the function of the MCU complex, MICU1 is critical for directing the assembly of the MCU complex in the presence of Ca^2+^ (Gottschalk et al., 2019). Interestingly, MICU1 is also critical for the induction of cold-induced cell death. In a cold-induced stress model, the deletion of MICU1 prevents hyperpolarization of the mitochondria, which in turn reduces lipid peroxidation and prevents subsequent ferroptosis (Nakamura et al., 2021).

In contrast to MICU1 and MICU2, MICU3 received little attention until recently, when it was found to modulate mitochondria specifically in brain tissue (Patron et al., 2019). Interestingly, the authors found that it interacted with MICU1 and formed disulfide bonds, which were similar to those between MICU1 and MICU2. However, unlike the case with MICU2, the expression of MICU3 was shown to lead to enhanced uptake of Ca^2+^ into the mitochondria during intensive neuronal activity, which is explained by a higher Ca^2+^ affinity of MICU3 than that of MICU2 (Figure 2). These findings led to the proposal that MICU3 functions similarly to MICU2 by forming heterodimers with MICU1 to control gating of the MCU complex, and that the MICU2/MICU3 ratio determines the Ca^2+^ sensitivity of the MCU complex (Patron et al., 2019; Garbincius and Elrod, 2022). Recent experiments showed that MICU3 is responsible for a higher sensitivity of mitochondrial Ca^2+^ uptake to cytosolic Ca^2+^ in neurons as compared to non-neuronal cells such e.g., HEK293 cells that likely lack MICU3 and rely on the MICU1-MICU2 complex to gate the MCU channel (Ashrafi et al., 2020). Notably, this MICU3-dependent sensitization of mitochondrial Ca^2+^ uptake plays a critical role as the synapses by boosting Ca^2+^-dependent ATP synthesis in presynaptic terminals (Ashrafi et al., 2020). Additional knowledge of the role of MICU3 within the MCU complex will be important for understanding the mechanism by which it influences neuronal activity, but the available data show that it is critical for normal neuronal activity.

### EMRE

Another important component of the uniporter complex is EMRE, which was named for its role as an essential MCU regulator (Sancak et al., 2013; Tsai et al., 2016). This protein is critical for normal uniporter function. It is a 10 kD protein with only one transmembrane domain. When EMRE is deleted the MCU pore forms, but it is not functional and it fails to interact with MICU1 and MICU2 (Sancak et al., 2013; Tsai et al., 2016). EMRE is encoded only in metazoan genomes with no homologs in other organisms (Sancak et al., 2013; Tsai et al., 2016; Liu et al., 2017). The MCU/EMRE complex assembles at a 1:1 stoichiometry, and it does so on the membrane facing side of the MCU tetramer within the transmembrane domains (Wang et al., 2019). This stoichiometry was further supported by recent identification of the assembled MCU complex with EMRE, MICU1, and MICU2 all present. Interestingly, the structure of the EMRE containing MCU complex is similar regardless of whether or not MICU proteins are bound (Fan et al., 2020). This shows that proper MCU/EMRE assembly is not dependent upon MICUs. Recently, EMRE KO mice were characterized and found to be viable and to have normal metabolism, however, the birth rates were low. The EMRE KO mice had reduced mitochondrial Ca^2+^ uptake and were resistant to Ca^2+^ driven mitochondrial dysfunction, but they were not protected from ischemic reperfusion injury (Liu et al., 2020). These mice will be a useful model for further determining the role of Ca^2+^ uptake *via* EMRE/MCU complex formation in other diseases in which mitochondrial Ca^2+^ overload occurs.

### Mitochondrial Ca^2+^ extrusion

Under physiological conditions, mitochondrial Na^+^/Ca^2+^ (mtNCX) and H^+^/Ca^2+^ (mtHCX) exchangers mediate Ca^2+^ extrusion from mitochondria (De Stefani et al., 2016). In excitable cells, such as neurons and muscles, mtNCX is the main contributor to this transport (Crompton et al., 1978; Thayer et al., 2002; Medvedeva et al., 2008; Jadiya et al., 2019; Rysted et al., 2021; Yang et al., 2021; Hagenston et al., 2022). It was discovered that when Ca^2+^ uptake was blocked, Ca^2+^ extrusion from the mitochondria occurs in a Na^+^ dependent manner (Nicholls, 1978). Interestingly, the release of Ca^2+^ by mitochondria was not inhibited by the inhibitor of Ca^2+^ uptake ruthenium red, suggesting that a reversal of the uptake mechanism is not responsible for the Ca^2+^ release (Rossi et al., 1973; Nicholls, 1978). An early study, performed in isolated mitochondria, identified the exchanger responsible for Ca^2+^ extrusion as 110 kD in size (Li et al., 1992).

In 2010, Sekler and colleagues proposed that a so-called Na^+^/Ca^2+^/Li^+^ exchanger (NCLX), a product of gene *Slc8b1*, represents an essential component of the mitochondrial Na^+^/Ca^2+^ exchanger (Palty et al., 2010). Historically, NCLX was first described as the sixth member of the family of Na^+^/Ca^2+^/K^+^ exchangers, and it was named NCKX6 (Cai and Lytton, 2004b). It was found to express as two splice isoforms, with the short isoform being localized to the plasma membrane and the full-length isoform being localized to the ER (Cai and Lytton, 2004b). At about the same time it was shown that NCKX6 was able to exchange Li^+^ for Ca^2+^ across the plasma membrane as effectively as it could exchange Na^+^ for Ca^2+^, and the protein was renamed to NCLX (Palty et al., 2004; Palty et al., 2006). Later, the same group showed that in addition to the plasma membrane and ER, NCLX was also enriched in the mitochondrial fraction (Palty et al., 2010). It was also found to partially localize to the mitochondria using gold-particle transmission electron microscopy, although some particles were located outside of the mitochondria as well. In addition to the localization data, these functional assays revealed that mitochondrial Ca^2+^ efflux increased when NCLX was overexpressed and that it decreased when NCLX was silenced using an shRNA approach (Palty et al., 2010). All this led to the proposal that NCLX is an essential contributor to the mitochondrial Na^+^/Ca^2+^ exchange (Palty et al., 2010).

NCLX is broadly expressed throughout the body including the brain and spinal cord (Palty et al., 2010; Jadiya et al., 2019; Patron et al., 2019). The *in vivo* role of NCLX was first demonstrated in the cardiomyocytes of adult mice (Luongo et al., 2017). Cardiomyocyte-specific deletion of NCLX resulted in severe lethality (87%) within two weeks, due to heart failure; whereas overexpression of NCLX resulted in protection from ischemic heart damage (Luongo et al., 2017). The same group also provided evidence for the role of NCLX in aging brain (Jadiya et al., 2019). They showed that in the 3xTg-AD mouse model of Alzheimer’s disease, the neuron-restricted deletion of NCLX increased tau and amyloid pathology and increased the rate of cognitive decline in mice. Restoration of NCLX expression reduced the acceleration of Alzheimer’s pathology (Jadiya et al., 2019). Similarly, disruption of NCLX expression was shown to sensitize hippocampal neurons to excitotoxic stress (Hagenston et al., 2022). A recent study has reported that a human recessive missense mutation in the gene encoding NCLX, *SLC8B1*, impairs its function and is associated with severe intellectual disability (Stavsky et al., 2021). The same study also demonstrated that deletion of NCLX in mice impaired synaptic transmission and long-term synaptic plasticity (Stavsky et al., 2021). Collectively, these studies highlight a key role of NCLX in regulating synaptic function and neuronal survival.

Despite the appreciation of NCLX as a mediator of Ca^2+^ extrusion from mitochondria, several studies showed that NCLX is also localized on the plasma membrane, endoplasmic reticulum (ER), and possibly other organelles (Haworth and Hunter, 1979; Bernardi, 1999; Cai and Lytton, 2004a; Palty et al., 2004; Palty et al., 2006; Han et al., 2015; Rysted et al., 2021). There is also inconsistency between the ability of NCLX to exchange Ca^2+^ for Li^+^ nearly as effectively as for Na^+^ (Palty et al., 2004; Roy et al., 2017) and the observations that Li^+^-driven mitochondrial Ca^2+^ efflux is only 10–25% of that driven by Na^+^ (Crompton et al., 1977; Tokunaga et al., 2000; Rysted et al., 2021). In addition, recent studies showed that NCLX knockdown or knockout had little effect on mitochondrial Ca^2+^ extrusion in airways smooth muscle and B lymphocytes, respectively (Emrich et al., 2022; Johnson et al., 2022). These findings raise several important questions for future research regarding the mechanisms of mitochondrial Ca^2+^ extrusion and the roles of NCLX, such as: 1) What are the mechanisms of cellular trafficking of NCLX? 2) What are the functions of NCLX on the plasma membrane, ER and possibly other organelles? 3) Are there additional yet to be identified molecules that mediate Na^+^/Ca^2+^ exchange across the mitochondrial membranes?

### Molecular identity of the mPTP

Although Ca^2+^ uptake into the mitochondria is critical for normal cellular function, the entry of excessive amount of Ca^2+^ into the mitochondria can result in Ca^2+^ overload and lead to activation of the mitochondrial permeability transition pore (mPTP); this phenomenon has been associated with many different disease states, and thus the mPTP is considered a promising target for therapeutic intervention (Duchen et al., 1993; Irwin et al., 2003; Du et al., 2008; Martin et al., 2009; Martin et al., 2014). Ca^2+^ dependent activation of the mPTP, in isolated mitochondria, results in the formation of a large non-selective ion channel that is permeable to solutes up to approximately 1,500 Da in size, which allows for the passage of internalized Ca^2+^ and many proapoptotic proteins out of the mitochondria (Haworth and Hunter, 1979; Bernardi, 1999; Pivovarova and Andrews, 2010; Jung et al., 2020). Prolonged opening of the mPTP results in the loss of ΔΨ_mt_, which in turn prevents the production of ATP within the mitochondria and leads to cell death (Bernardi, 1999; Briston et al., 2019). Transient, low-conductance opening or “flickering” of the mPTP has been shown to occur in response to increased [Ca^2+^]_mt_ and helps to reduce the accumulation of Ca^2+^ into the mitochondria (Bernardi and Petronilli, 1996; Ichas et al., 1997; Bernardi, 1999; Loupatatzis et al., 2002; Rasola and Bernardi, 2011; Garbincius and Elrod, 2022). In further support of the Ca^2+^ dependent activation of mPTP, preventing Ca^2+^ uptake into the mitochondria by deleting MCU reduces mPTP activation in response to high levels of Ca^2+^ (Hamilton et al., 2021). Despite the presence of the mPTP being known for many years, the identity of the proteins that make up the mPTP remains highly debated.

Early hypotheses proposed that the mPTP could form as the result of contact between inner and outer mitochondrial proteins, specifically between voltage-dependent anion-selective channels (VDAC) on the outer membrane and adenine nucleotide translocators (ANT) on the inner mitochondrial membrane (Zamzami and Kroemer, 2001). Although, deletion of each of these proteins failed to inhibit formation of the mPTP (Giorgio et al., 2018). More recently, compelling evidence has pointed towards a role for the F_0_F_1_ ATP synthase in forming the mPTP (Giorgio et al., 2013; Alavian et al., 2014; Giorgio et al., 2018; Mnatsakanyan et al., 2019; Neginskaya et al., 2019; Carrer et al., 2021). Using blue native gel electrophoresis and immunoprecipitation, Giorgio and colleagues found that the essential mPTP regulator cyclophilin D interacts with F_0_F_1_ ATP synthase (Giorgio et al., 2009). Subsequent immunoprecipitation experiments identified that the oligomycin sensitivity conferring protein (OSCP) subunit of ATP synthase specifically interacts with cyclophilin D through the same binding site as the ATP synthase inhibitor benzodiazepine 423 (Stelzer et al., 2010; Giorgio et al., 2013). Following these findings, experiments suggested that the c-subunit of F_0_ was able to form a voltage-sensitive channel that also appeared to be sensitive to Ca^2+^ exposure (Alavian et al., 2014), and had conductance consistent with activated mPTP (Mnatsakanyan et al., 2022). When c-subunit expression was reduced using an shRNA, mPTP activation was reduced and the survival of HeLa cells increased (Alavian et al., 2014). The deletion of the c-subunit from HeLa cells prevented mPTP activation in response to ionomycin as efficiently as the pre-application of cyclosporin A and overexpression of the c-subunit increased the rate of mPTP transition (Bonora et al., 2013). In HAP1 cells, knocking out the c-subunit of F_0_ was also shown to significantly alter, but not eliminate, mPTP activity. In this context, channels formed and remained sensitive to the mPTP inhibitor cyclosporine A and to the ANT inhibitor, bongkrekate (Neginskaya et al., 2019). Deletion of specific subunits of the ATPase impaired the formation of the mPTP. Specifically, the deletion of the g subunit, which also reduced subunit e expression, impaired mPTP channel opening in both mitoplasts and in HeLa cells (Carrer et al., 2021). However, some studies suggested that the mPTP is not ATP synthase, by demonstrating mPTP activation even after the deletion of ATP synthase subunits (He et al., 2017a; He et al., 2017b; Carroll et al., 2019). In support of these findings, the deletion of the b and OSCP subunits in HAP1 cells did not interfere with functional mPTP currents that were not blocked by cyclosporin A (Carrer et al., 2021). It is possible that in the absence of ATPase subunits, the ANT could form a functional mPTP, consistent with the finding that addition of bongkrekate impaired mPTP opening in these cells (Neginskaya et al., 2019; Carrer et al., 2021). However, a recent study using intact HAP1 cells suggests that both ANT and ATPase are required for mPTP activation, but that mitochondrial depolarization occurs through low-conductance, cyclosporin A sensitive permeability transition (Neginskaya et al., 2022). These findings suggest that while mPTP activation requires both ANT and ATPase, the low-conductance or “flickering” activity may occur through either of these molecules or through different mechanisms altogether.

Collectively, the available data on the mPTP represent compelling evidence that the F_0_F_1_ ATPase plays a critical role in its formation, but also suggest that certain subunits of the ATPase can be compensated for by other mechanisms. The combination of the results for and against the notion that F_0_F_1_ ATPase is the mPTP suggest that cell-type specific differences exist and that the mPTP can form *via* several mechanisms (He et al., 2017a; He et al., 2017b; Carrer et al., 2021). There are also other proposed Ca^2+^ sensitive, pore-forming components of mPTP including the mitochondrial phosphate carrier (PiC) (Leung et al., 2008) and spastic paraplegia 7 (SPG7) (Shanmughapriya et al., 2015). However other studies suggest these proteins may have a regulatory role as opposed to a role in forming the pore (Briston et al., 2019; Hurst et al., 2019; Boyenle et al., 2022). The key cytotoxic role of mPTP in many diseases, makes this molecular complex an attractive therapeutic target for neuroprotection, and more generally, cytoprotection. However, the existence of compensatory mechanisms helps explain why targeting this system for the development of therapeutics is so challenging. Even if additional potential inhibitors are identified, they may have significant side effects given the importance of the ATPase for normal cellular function.

## Mitochondrial Ca^2+^ cycling in neuronal function

### Regulation of synaptic transmission

Ca^2+^ signaling plays an especially important role in neurons because it regulates many aspects of neurotransmitter release and synaptic function (Neher and Sakaba, 2008) (Figure 1). For example, Ca^2+^ triggers synchronous transmitter release *via* the low-affinity presynaptic Ca^2+^ sensors synaptotagmin 1, 2, and 9 (Xu et al., 2007; Bacaj et al., 2013). In addition, the subsequent endocytotic retrieval of synaptic vesicles is regulated by Ca^2+^- and calcineurin-dependent dephosphorylation of dynamin-1 and other endocytotic proteins (Cousin and Robinson, 2001; Clayton and Cousin, 2009). Presynaptic Ca^2+^ also regulates short-term synaptic plasticity by controlling the size of the readily releasable pool (RRP) of synaptic vesicles.

Presynaptic terminals are enriched for mitochondria, an observation that reflects high energy demand at the site of transmitter release (De Camilli et al., 2001; Hollenbeck, 2005; Wimmer et al., 2006). Ca^2+^ buffering by mitochondria limits the amplitude of the activity-driven elevation of presynaptic [Ca^2+^]_i_ and regulates synaptic strength at many different synapses, including central synapses, nerve-muscular junction, and the first sensory synapse (Tang and Zucker, 1997; David, 1999; David and Barrett, 2000; Billups and Forsythe, 2002; David and Barrett, 2003; Lee et al., 2007; Shutov et al., 2013; Kwon et al., 2016). Indeed, inhibition of mitochondrial Ca^2+^ uptake resulted in a larger presynaptic [Ca^2+^]_i_ elevation, an increase in the frequency of asynchronous release, and accelerated synaptic depression at the neuro-muscular junction (David, 1999; David and Barrett, 2000; 2003). Similar effects were described at the central synapses (Billups and Forsythe, 2002; Lee et al., 2007). In the case of GABAergic synaptic transmission between retinal amacrine cells, mitochondrial depolarization led to a reduction in evoked transmitter release that was attributed to an increase in Ca^2+^-dependent inactivation of presynaptic voltage-gated Ca^2+^ channels in the absence of Ca^2+^ buffering by mitochondria (Medler and Gleason, 2002).

Mitochondrial Ca^2+^ transport also contributes to post-tetanic potentiation (PTP) at neuro-muscular and central synapses (Tang and Zucker, 1997; Zhong et al., 2001; Storozhuk et al., 2005; García-Chacón et al., 2006; Lee et al., 2007). PTP is a form of short-term synaptic plasticity that is induced by high-frequency (tetanic) stimulation of the presynaptic terminal, and is characterized by an enhancement of synaptic strength that lasts for many minutes (Zucker and Regehr, 2002). This phenomenon relies on a prolonged presynaptic [Ca^2+^]_i_ elevation (residual [Ca^2+^]_i_ or [Ca^2+^]_i_ plateau) following the initial [Ca^2+^]_i_ rise to the micromolar levels in response to intense stimulation (Zucker and Regehr, 2002; Neher and Sakaba, 2008). In many instances prolonged Ca^2+^ release from presynaptic mitochondria accounts for the residual [Ca^2+^]_i_ (Tang and Zucker, 1997; Zhong et al., 2001; García-Chacón et al., 2006; Lee et al., 2007; Medvedeva et al., 2008). For example, high-frequency presynaptic stimulation produced a prolonged residual presynaptic [Ca^2+^]_i_ elevation and PTP in nerve muscular junctions of crayfish and mouse, as well at mossy fiber synapses of the rat hippocampus that were blocked by inhibitors of mitochondrial Ca^2+^ transport (Tang and Zucker, 1997; Zhong et al., 2001; García-Chacón et al., 2006; Lee et al., 2007). A similar mechanism was shown to contribute to short-term facilitation at the calyx of Held synapse (Yang et al., 2021). Mitochondria-dependent PTP was also reported for GABAergic synaptic transmission between neocortical neurons, although presynaptic [Ca^2+^]_i_ was not measured in this study (Storozhuk et al., 2005). It is most likely that Ca^2+^ release from presynaptic mitochondria is mediated by the mitochondrial Na^+^/Ca^2+^ exchanger (García-Chacón et al., 2006; Lee et al., 2007; Medvedeva et al., 2008). However alternative mechanisms may also be involved at some synapses (Zhong et al., 2001).

Findings from recent studies in *Drosophila* motor terminals suggest that mitochondrial Ca^2+^ uptake enhances energy metabolism by presynaptic mitochondria (Chouhan et al., 2012; Ivannikov and Macleod, 2013). These observations are consistent with the ability of mitochondrial Ca^2+^ to stimulate Ca^2+^-dependent dehydrogenases, pyruvate dehydrogenase, isocitrate dehydrogenase and α-ketoglutarate dehydrogenase, thereby increasing the input of critical substrates into the oxidative phosphorylation reaction and ATP synthesis (Denton, 2009; Glancy and Balaban, 2012). Given that [Ca^2+^]_mt_ rarely exceeds 5 μM, it is likely that pyruvate dehydrogenase and α-ketoglutarate dehydrogenase, each of which has Ca^2+^ K_d_ of ∼0.8 μM (for comparison, isocitrate dehydrogenase has a Ca^2+^ K_d_ of∼40 μM) (McCormack et al., 1990), are the primary targets of matrix Ca^2+^. Such a mechanism may be an important coordinator of ATP supply and demand at the synapse. Indeed, increased synaptic activity is associated with larger amounts of Ca^2+^ entering the presynaptic terminal, which in turn increases Ca^2+^ flux into the mitochondria and stimulates ATP-synthesis. Thus, mitochondrial Ca^2+^ transport regulates synaptic transmission through at least two general mechanisms, with one contributing to the shaping of presynaptic [Ca^2+^]_i_ signals, and the other impacting the ATP synthesis at the synapse.

Recent work started exploring the roles of specific molecular components of the mitochondrial Ca^2+^ transport in synaptic transmission. Inhibiting MCU in the *Drosophila* mushroom body neurons caused impairment of synaptic function and olfactory memory (Drago and Davis, 2016). In rat hippocampal neurons, MCU and MICU3 were found to contribute to presynaptic ATP synthesis during synaptic activity (Ashrafi et al., 2020). The same study also found that MCU silencing slowed the rate of endocytosis of synaptic vesicles (Ashrafi et al., 2020). However, an opposite effect of MCU silencing on the rate of synaptic vesicle endocytosis in hippocampal neurons was also reported (Marland et al., 2016). Thus, despite recent progress many questions remain about the role of MCU and other components of the MCU complex at synapses, including their specific roles in regulating synaptic transmission and synaptic plasticity at various excitatory and inhibitory synapses.

### Regulation of intrinsic excitability in neurons

One of the essential roles of Ca^2+^ as a second messenger is to regulate neuronal excitability (Figure 1). This is achieved through the gating of three types of ion channels: Ca^2+^-activated K^+^, Ca^2+^-activated Cl^−^ and Ca^2+^-activated cation channels (Partridge and Swandulla, 1988; Marty, 1989; Scott et al., 1995; Sah, 1996; Hartzell et al., 2005). The small- and large-conductance Ca^2+^-activated K^+^ channels, SK and BK, respectively, are widely expressed in neurons and play important roles in regulating their excitability (Sah, 1996; Wei et al., 2005). Ca^2+^-activated cation and Cl^−^ channels require [Ca^2+^]_i_ elevations in the 0.1–1 μM range to open, and the former channels contribute to neuronal depolarization, whereas the latter can either depolarize or hyperpolarize the membrane depending on the transmembrane Cl^−^ gradient and membrane potential (Partridge and Swandulla, 1988; Scott et al., 1995; Hartzell et al., 2005). Ca^2+^ also modulates many other channels and receptors including voltage-gated Ca^2+^ channels, TRP channels, and NMDA glutamate receptors (McBain and Mayer, 1994; Catterall, 2000; Caterina and Julius, 2001; Budde et al., 2002; Clapham, 2003; Chuang et al., 2004; Catterall et al., 2013).

Mitochondria buffering and the release of Ca^2+^ have complex effects on Ca^2+^-activated and -modulated conductances and neuronal excitability. For example, blocking mitochondrial Ca^2+^ uptake by applying protonophores or inhibitors of electron transport resulted in hyperpolarization mediated by Ca^2+^-activated K^+^ channels in hippocampal and myenteric neurons (Nowicky and Duchen, 1998; Vanden Berghe et al., 2002). Similarly, intracellular application of the MCU selective inhibitor Ru360 facilitated slow afterhyperpolarization (AHP) in CA1 hippocampal pyramidal neurons (Groten and MacVicar, 2022). Several reports also showed that blocking mitochondrial Ca^2+^ uptake reduced voltage-gated Ca^2+^ currents, likely due to an increase in Ca^2+^-dependent inactivation of the Ca^2+^ channels (Hernandez-Guijo et al., 2001; Medler and Gleason, 2002; Shutov et al., 2013). Thus, under normal conditions, mitochondrial Ca^2+^ uptake limits Ca^2+^-dependent activation of BK and SK channels, and minimizes Ca^2+^-dependent inactivation of voltage-gated Ca^2+^ channels. The latter effect is especially critical for the synapse, where Ca^2+^ flux through these channels trigger transmitter release.

A recent study has demonstrated a specific role of MCU in regulating so-called Ca^2+^-release activated Ca^2+^ (CRAC) channels and Ca^2+^ signaling (Yoast et al., 2021). CRAC channels are activated in response to agonist stimulation and inositol-1,4,5-trisphosphate-induced Ca^2+^ release from ER stores and play important roles in Ca^2+^ signaling in a broad variety of non-excitable and excitable cells (Prakriya and Lewis, 2015; Emrich et al., 2021). In neurons, CRAC channels can control both excitability and synaptic transmission (Gemes et al., 2011; Ureshino et al., 2019; Hori et al., 2020; Maneshi et al., 2020). In their work, Yoast et al., found that MCU deletion led to increased Ca^2+^-dependent inactivation of CRAC channels and reduced Ca^2+^ currents through the channels. Yet, the net result was an overall amplification of cytosolic Ca^2+^ signaling caused by elimination of mitochondrial Ca^2+^ buffering in the absence of MCU (Yoast et al., 2021; Walters and Usachev, 2022). Although this effect was documented in non-excitable cell lines, given that CRAC channels and MCU are present in the nervous tissues, it is predicted that a similar mechanism could contribute to the regulation of neuronal excitability (Walters and Usachev, 2022).

## Mitochondrial Ca^2+^ cycling in neurological diseases

### Mitochondrial Ca^2+^ regulation in epilepsy

Epilepsy is one of the most common neurologic conditions worldwide, with close to 70 million people currently diagnosed and approximately 1 in 26 people expected to develop epilepsy in their lifetime (Hesdorffer et al., 2011; Friedman et al., 2018). For many of these patients, available pharmaceutical interventions reduce the prevalence of seizures. Unfortunately, 30% of patients have epilepsy that is poorly controlled by current pharmaceuticals, a condition referred to as refractory epilepsy (Kwan and Brodie, 2000). Patients with refractory epilepsy are at a much greater risk of experiencing sudden unexpected death in epilepsy (SUDEP, 1/150) than those with non-refractory epilepsy (1/1,000) (Thurman et al., 2014; Friedman et al., 2018). The increased risk of mortality associated with refractory epilepsy justifies further research of mechanisms underlying epilepsy; such knowledge will be required to identify novel therapeutic targets for epilepsy.

The changes in neuronal networks that result in epilepsy can have multiple causes. For example, lesions that occur following strokes or traumatic brain injuries result in structural changes that increase the risk of epilepsy. Genetic mutations are another common cause for epilepsy. In many cases, however, epilepsy is of unknown etiology, i.e., no clear underlying cause can be identified (Berg et al., 2010). Multiple forms of epilepsy have been associated with genetic mutations that lead to metabolic and mitochondrial dysfunction; mutations in both mitochondrial and nuclear DNA have been implicated in these cases (Bindoff and Engelsen, 2012; Zsurka and Kunz, 2015; Pearson-Smith and Patel, 2017). One such example is myoclonic epilepsy and ragged-red fibers (MERRF) syndrome, for which myoclonus is a defining feature that presents along with other neurologic defects. Studies of cultured cells into which the patient mitochondrial DNA mutation had been introduced showed that this resulted in decreased ATP-dependent protease activity and increased ROS production (Wu et al., 2010). MERRF mutations also resulted in disrupted mitochondrial Ca^2+^ handling but did not alter cytosolic Ca^2+^ regulation. The authors observed reduced ATP production in these cells following stimulation with histamine. They hypothesized that the reduction in ATP production resulted from a reduction in Ca^2+^ entry into the mitochondria, and that this led to reduced activation of Ca^2+^-dependent enzymes (Brini et al., 1999). Other examples of genetic diseases that lead to epilepsy by interfering with metabolic function include Alpers-Huttenlocher syndrome (AHS) and Leigh syndrome; in each, mutations lead to reduced ATP production and refractory epilepsy (Bindoff and Engelsen, 2012; Zsurka and Kunz, 2015; Pearson-Smith and Patel, 2017). Although, disrupted energy production results in many different forms of epilepsy, a loss-of-function mutation in MICU1 in humans instead results in myopathy, movement disorders, and learning disabilities (Logan et al., 2014). The propensity for mitochondrial diseases to result in epilepsy identify the mitochondria as targets for new therapies for epilepsy.

**FIGURE 3:**
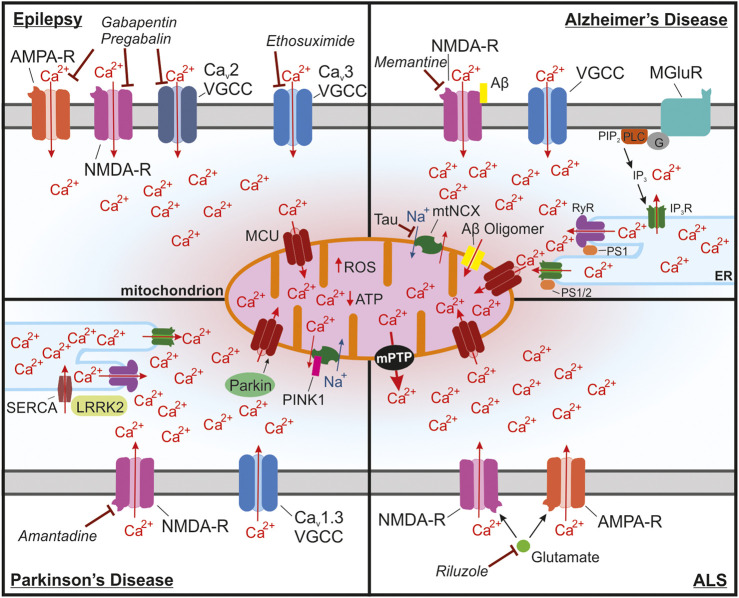
Mitochondrial Ca^2+^ overload leading to activation of the mPTP is a common pathway in many neurological diseases. **(A)** In epilepsy, seizures lead to buildup of cytosolic and mitochondrial Ca^2+^ through NMDA receptors, Ca^2+^ permeable AMPA receptors and Ca_v_2 and Ca_v_3 voltage-gated Ca^2+^ channels (VGCCs) in neurons that are in a state of hyperexcitability. Long seizures or repeated seizures can lead to Ca^2+^ overload in the mitochondria and trigger activation of the mPTP, ultimately leading to neuronal toxicity. Commonly prescribed antiepileptic drugs target these channels including: ethosuximide, which is a Ca_v_3 VGCC inhibitor, as well as gabapentin and pregabalin, which act by binding to the with α2δ-1 subunit of voltage-gated Ca^2+^ channels, which could disrupt plasma membrane expression and function of Ca_v_2 VGCCs, NMDA receptors, and Ca^2+^ permeable AMPA receptors. **(B)** In Alzheimer’s disease, Ca^2+^ overload in the mitochondria occurs through multiple mechanisms. Ca^2+^ entry into the cytosol can be enhanced by binding of Aβ to NMDA receptors. Internal release of Ca^2+^ from the ER through activation of metabotropic glutamate receptors (mGluR) also increases the accumulation of Ca^2+^ into the mitochondria. Enhanced Ca^2+^ extrusion from the ER has also been shown to occur through interactions between presenilin 1/2 (PS1/2) and inositol trisphosphate receptors (IP_3_R) and ryanodine receptors (RyR). This interaction seems to occur at mitochondria associated membranes (MAMs), which due to the proximity to the mitochondria, drives Ca^2+^ uptake into the mitochondria. Aβ oligomers also can form a pore in the mitochondria that can lead to increases in Ca^2+^ in the mitochondria. Ca^2+^ extrusion through the mitochondrial Na^+^/Ca^2+^ exchanger (mtNCX) has been shown to be impaired by tau accumulation leading to excessive Ca^2+^ accumulation in the mitochondria. Memantine, one of the FDA approved drugs for AD, inhibits NMDA receptors, which could block excessive Ca^2+^ uptake and to reduce glutamate induced excitotoxicity. **(C)** In Parkinson’s disease (PD), substantia nigra dopaminergic neurons are at risk of Ca^2+^ overload due to the presence of voltage-gated Ca_v_1.3 channels, which are critical for the pacemaking activity of these neurons. One common form of autosomal recessive familial PD occurs through mutations in *PARK2*, the gene for Parkin. Parkin is responsible for regulating mitochondrial Ca^2+^ uptake isoform 1 (MICU1) degradation and may lead to disrupted MCU assembly altering mitochondrial Ca^2+^ uptake. Another form of autosomal recessive PD is caused by mutations in *PINK1*, which can impair mtNCX function and increase Ca^2+^ accumulation into the mitochondria. In astrocytes, ER Ca^2+^ release at MAMs may be enhanced in PD due to mutations in leucine-rich repeat kinase 2 (LRRK2), which enhance Ca^2+^ uptake into the ER through activation of sarcoplasmic reticulum Ca^2+^ -ATPase (SERCA). The NMDA receptor antagonist amantadine is used to prevent dyskinesia in PD patients. **(D)** In amyotrophic lateral sclerosis (ALS), motor neurons of the spinal cord and brain begin to degenerate. These neurons have been shown to have increased Ca^2+^ uptake through glutamate NMDA and Ca^2+^ permeable AMPA receptors, which enhances their susceptibility to mitochondrial Ca^2+^ overload. Excitotoxic glutamate, which signals through NMDA and GluA2-lacking Ca^2+^ permeable AMPA receptors, is the target of Riluzole, one of the few FDA approved drugs for ALS.

Epilepsy occurs when brain networks transition into a state of hyperexcitability, a process known as epileptogenesis (Scharfman, 2007). Hyperexcitability of neuronal networks is a key change that occurs in individuals who develop epilepsy. It can be caused by changes in gene expression, posttranslational modification, or synaptic circuits (Kinjo et al., 2018). These processes are frequently Ca^2+^ dependent. For example, dendritic arborization and synaptogenesis are dependent upon changes in gene expression following increases in intracellular Ca^2+^ levels (Lohmann and Wong, 2005; Redmond and Ghosh, 2005). Mitochondrial Ca^2+^ regulation has been shown to be important for neurotransmission and altered mitochondrial function can disrupt synaptogenesis (MacAskill et al., 2010; Sun et al., 2013). Proper Ca^2+^ regulation by mitochondria is essential for normal neuronal processes including synaptogenesis, excitability, neurotransmission, and ATP synthesis (Billups and Forsythe, 2002; Thayer et al., 2002; Mattson et al., 2008; Usachev, 2015). During seizures, excess Ca^2+^ can build up within the cytosol and eventually lead to mitochondrial Ca^2+^ overload, which is known to lead to cell death (Figure 3).

Indeed, hippocampal sclerosis and neuronal loss in the hippocampus and other limbic and mesial cortical structures is the very common pathological finding in patients with temporal lobe epilepsy, the most prevalent form of epilepsy in humans (Levesque and Avoli, 2013; Tai et al., 2018). In fact, frequent generalized convulsive seizures are a strong predictor of cognitive decline as well as progressive development of behavioral and psychiatric abnormalities in patients with epilepsy, all of which is linked to neuronal damage in the limbic and mesial cortical regions (Kovac et al., 2017; Tai et al., 2018; Farrell et al., 2019). Cell death is particularly apparent following status epilepticus, sustained generalized convulsive seizures lasting 5 min or more (Walker, 2018). These prolonged seizures result in Ca^2+^ overload in neurons and astrocytes (Tian et al., 2005; Fellin et al., 2006; Ding et al., 2007). Neurotoxic build-up of glutamate released from firing neurons and astrocytes is thought to be the major trigger of neuronal damage (i.e., excitotoxicity) caused by prolonged seizures, as well as in other pathological conditions (e.g., stroke, and neurodegenerative diseases) (Nicholls and Ward, 2000; Mattson et al., 2008; Moskowitz et al., 2010; Kovac et al., 2017). Glutamate-induced Ca^2+^ entry, largely *via* NMDA as well as Ca^2+^-permeable AMPA glutamate receptors, mitochondrial Ca^2+^ overload and Ca^2+^ deregulation initiate neurotoxic cascades through many mechanisms, including rupture of mitochondrial membranes, mitochondrial depolarization, ROS generation and opening of mitochondrial permeability transition pore (mPTP) and release of cytochrome c and other proapoptotic factors (Figure 3) (Nicholls and Ward, 2000; Newmeyer and Ferguson-Miller, 2003; Orrenius et al., 2003; Munoz-Pinedo et al., 2006; Mattson et al., 2008; Moskowitz et al., 2010; Guo and Ma, 2021). Accordingly, selective antagonists of glutamate NMDA or Ca^2+^-permeable AMPA receptors reduced seizure-induced neuronal toxicity in animal models of status epilepticus and temporal lobe epilepsy (Ding et al., 2007; Kovac et al., 2017; Walker, 2018; Konen et al., 2020).

Antiepileptic drugs such as gabapentin and pregabalin were also shown to reduce hippocampal neuronal loss in the rodent models of temporal lobe epilepsy and to improve mitochondrial function (Huang et al., 2013; Rossi et al., 2013; Finsterer and Scorza, 2017). Although originally designed as GABAmimetics, it is now well accepted that the anticonvulsant action of these gabapentinoids is mediated by mechanisms distinct from GABAergic activity. Instead, it has been proposed that these drugs interact with α2δ-1 subunit of voltage-gated Ca^2+^ channels, which in turn interferes with α2δ-1-dependent insertion and retention of α1 subunits of the channels (Dolphin, 2012; Sills and Rogawski, 2020) (Figure 3). An additional mechanism likely involves interaction of α2δ-1 subunit with glutamate NMDA receptor (Sills and Rogawski, 2020), and disrupting this interaction by gabapentin was shown to inhibit neuronal toxicity caused by ischemic stroke (Luo et al., 2018). The α2δ-1 subunit can also interact with the GluA1 and GluA2 subunits of glutamate AMPA receptors, which promotes expression of GluA2-lacking Ca^2+^-permeable AMPA receptors, the process that can be disrupted by gabapentin (Li et al., 2021). Thus, it is plausible that the described neuroprotective effects of gabapentin and pregabalin are mediated by their inhibitory action on NMDA and Ca^2+^-permeable GluA2-lacking AMPA receptors (Figure 3).

The mitochondrial Ca^2+^ overload due to mitochondrial Ca^2+^ uptake is a major trigger of excitotoxicity and neuronal death downstream of Ca^2+^ entry into neurons *via* glutamate NMDA receptors and other Ca^2+^ channels (Nicholls and Ward, 2000; Mattson et al., 2008; Kovac et al., 2017). Consequently, it is predicted that inhibiting mitochondrial Ca^2+^ uptake is neuroprotective. To this end, a recent study reported that systemically administered MCU inhibitor Ru360 reduced hippocampal neuronal death in a rat model of pilocarpine-induced status epilepticus (Wang et al., 2015). The findings of this work however, should be interpreted with caution because Ru360 poorly penetrates through the plasma membrane, which is required for inhibiting MCU (Hajnoczky et al., 2006; Woods and Wilson, 2020; Marta et al., 2021). In addition, Ru360 is very unstable for *in vivo* work, and can affect various ion channels of the plasma membrane when applied extracellularly (Hajnoczky et al., 2006; Woods and Wilson, 2020; Marta et al., 2021). Thus, additional studies are needed to test the specific roles of the mitochondrial Ca^2+^ uptake and release mechanisms transport in neurotoxicity associated with seizures and epilepsy.

In summary, mitochondrial Ca^2+^ cycling can affect neural activity and susceptibility to seizures and epilepsy *via* several mechanisms, including regulation of neuronal excitability, synaptic transmission, bioenergetics, and excitotoxicity (Figures 1, 3). A better understanding of neuronal Ca^2+^ regulation may prove essential for further elucidation of the mechanisms that underlie epilepsy, and thus for identifying potential therapies.

### Mitochondrial Ca^2+^ regulation in Alzheimer’s disease

Alzheimer’s disease (AD) is a debilitating neurodegenerative disorder and the most common form of dementia in the elderly. It is characterized by progressive impairment of memory and cognitive functions and may lead to a completely vegetative state and early death (Querfurth and LaFerla, 2010; Scheltens et al., 2016; Corriveau et al., 2017). AD currently affects approximately 5.8 million Americans who are 65 years of age and older and current estimates predict that this number will increase to 13.8 million by 2050 (Alzheimer's and Dementia, 2020a).

AD pathology is characterized by an accumulation of β-amyloid (Aβ) and the formation of neurofibrillary tangles from accumulated tau protein (Hyman et al., 2012). According to the amyloid hypothesis of AD, progressive accumulation and deposition of Aβ42 plaques leads to a cascade of pathological changes in the brain that ultimately lead to the synaptic and neuronal loss and progressing dementia (Selkoe and Hardy, 2016; Golde et al., 2018). Such changes include inflammation, the activation of microglia and astrocytes, an accumulation of hyperphosphorylated tau and neurofibrillary tangles, altered ionic homeostasis, and oxidative injury (Selkoe and Hardy, 2016; Golde et al., 2018). Neuronal Ca^2+^ deregulation is thought to play critical roles during various phases of AD and can manifest in many forms, including chronic elevation of the cytosolic and mitochondrial Ca^2+^ concentrations, enhanced Ca^2+^ release from intracellular Ca^2+^ stores, spontaneous Ca^2+^ spikes, and enhanced activity of glutamate NMDA receptors (Bezprozvanny and Mattson, 2008; Mattson et al., 2008; Querfurth and LaFerla, 2010; Workgroup and Khachaturian, 2017) (Figure 3). Although various mechanisms contribute to Ca^2+^ deregulation in AD, most of them ultimately lead to a common outcome–mitochondrial Ca^2+^ overload. This in turn, is thought to trigger neuronal demise through a range of neurotoxic mechanisms, including mPTP opening and release of proapoptotic factors, mitochondrial depolarization and ROS generation (Bezprozvanny and Mattson, 2008; Mattson et al., 2008; Querfurth and LaFerla, 2010).

Mitochondrial dysfunction in AD may be driven by multiple factors, but in this review we focus on the role of Ca^2+^ dysregulation in the progression of AD. Ca^2+^ dysregulation by mitochondria in the context of AD pathology was proposed to be a consequence of the aggregation of Aβ within the mitochondria by forming functional channels that have a high affinity for Ca^2+^ ions (Figure 3) (Arispe et al., 1993; Jang et al., 2007; Hansson Petersen et al., 2008; Rosales-Corral et al., 2012). Direct channel formation into the mitochondria has received limited attention since, but Aβ induced Ca^2+^ release from ER has been proposed to contribute to mitochondrial Ca^2+^ overload (Jensen et al., 2013). In both *Xenopus* oocytes and cultured SH-SY5Y cells, ER Ca^2+^ release was triggered by soluble Aβ *via* generation of inositol triphosphate (IP_3_) (Demuro and Parker, 2013; Jensen et al., 2013). Interestingly, in SH-SY5Y cells an increase in ER Ca^2+^ was also observed in the absence of IP_3_ receptors, suggesting that a more direct effect of Aβ on the ER accounts for the increased cytotoxicity of Aβ oligomers (Jensen et al., 2013). ER Ca^2+^ regulation is also disrupted by mutations in presenilin 1 (PS1) and presenilin 2 (PS2). Mutated PS1 and PS2 modulate IP_3_R activity and increase Ca^2+^ release (Cheung et al., 2008; Modesti et al., 2021). PS1 mutants have also been shown to interact with ryanodine receptors and increase their activity (Rybalchenko et al., 2008; Chakroborty et al., 2009; Modesti et al., 2021), which combined with increased release from IP3-sensitive Ca^2+^ stores might contribute to elevated cytosolic Ca^2+^, excessive mitochondrial Ca^2+^ load, and Ca^2+^ dysregulation associated with AD (Figure 3). The store-operated Ca^2+^ entry into the ER is also disrupted in “aging” neurons and is further deregulated by the presence of Aβ oligomers, which drives increases in cytosolic Ca^2+^ concentration (Calvo-Rodriguez et al., 2019). Injections of Aβ oligomers into the rat hippocampus leads to increased expression of type 2 ryanodine receptors at mitochondria associated membranes, which leads to increased Ca^2+^ release from the ER in response to Ca^2+^ signals and drives increased cytosolic Ca^2+^ concentrations (More et al., 2018). In addition to its potential direct actions on internal Ca^2+^ stores, Aβ is thought to modulate NMDA receptor activity and has been shown to contribute to Ca^2+^-induced cytotoxicity (Popugaeva et al., 2018). Glutamate induced excitotoxicity through NMDA receptors is also the target of one of the few FDA approved therapies for AD, memantine (Sonkusare et al., 2005). However, memantine’s effectiveness has been limited to symptomatic improvements in cognition of patients with moderate-to-severe AD (Areosa et al., 2005; Li et al., 2019).

It has been proposed that many forms of Ca^2+^ dysregulation in AD and other neurodegenerative disorders ultimately converge on mitochondrial Ca^2+^ overload, a crucial event that triggers mPTP opening, release of proapoptotic factors, ROS generation, and cell death (Bezprozvanny and Mattson, 2008; Mattson et al., 2008; Plotegher et al., 2021). Consequently, inhibiting MCU is predicted to be neuroprotective. Recent studies support this idea. Indeed, MCU was shown to be upregulated in the brain of 3xTg-AD (APP/PS1/tau model of AD) mice, whereas knocking down MCU led to reduced synaptic loss, increased mitochondrial mass and improved behavioral performance in memory tasks in 3xTg-AD mice (Cai et al., 2022). Another group recently reported that application of soluble Aβ peptides onto neurons increased mitochondrial Ca^2+^ concentration and promoted neuronal death, which could be reversed by extracellularly applied MCU inhibitor Ru360 (Calvo-Rodriguez et al., 2020). Complicating the interpretation of these findings is the observations that Ru360 poorly penetrates through the plasma membrane, which is required for inhibiting MCU, and can also affect various ion channels on the plasma membrane (Hajnoczky et al., 2006; Woods and Wilson, 2020; Marta et al., 2021). Thus, additional studies using more suitable pharmacological tools and genetic approaches will be needed to thoroughly test the role of MCU and other components of the MCU complex in AD models.

Along with Ca^2+^ uptake machinery, mitochondrial Ca^2+^ extrusion mechanisms are also potentially critical in AD progression. Recently, tau has been shown to impair Ca^2+^ extrusion out of the mitochondria through mtNCX and resulted in faster mitochondrial depolarization in response to repeated stimulations (Britti et al., 2020; Esteras and Abramov, 2020). NCLX, which has been proposed to be molecular determinant of the mtNCX transport, has reduced expression in the frontal cortex of patients with AD and overexpression of NCLX in animal models of AD slows disease progression (Jadiya et al., 2019; Garbincius and Elrod, 2022). While Ca^2+^ cycling within the cytosol, ER, and mitochondria are key factors in AD progression (Figure 3), further investigation is required to identify how the relationship between these cellular machineries is driving Ca^2+^ overload, mitochondrial dysfunction, and apoptotic signaling.

### Mitochondrial Ca^2+^ regulation in Parkinson’s disease

Parkinson’s disease (PD) is a progressive neurodegenerative disorder that is defined by the loss of dopaminergic neurons in the substantia nigra pars compacta (SNpc) (Braak et al., 2003; Bezprozvanny, 2009; Surmeier et al., 2017; Fu et al., 2018). Before the substantia nigra deteriorates, neurons in multiple brainstem nuclei (including the dorsal motor nucleus, the raphe nucleus, and the locus coeruleus) begin to die (Braak et al., 2003; Fu et al., 2018). Another common hallmark of PD is Lewy body pathology, which spreads to some common brain regions as the disease progresses (Surmeier et al., 2017). Despite the variability of Lewy pathology, one common trend occurs, which is the neuronal loss within the SNpc (Surmeier, 2007). Dopaminergic neurons of the SNpc are uniquely susceptible to perturbations that lead to the development of PD, when compared to dopaminergic neurons of the ventral tegmental area (VTA), which has been shown due to the dependence of SNpc neurons on Ca_v_1.3 for their rhythmicity (Chan et al., 2007; Surmeier, 2007; Chan et al., 2009; Fu et al., 2018). This unique dependence on Ca_v_1.3 likely leads to greater accumulation of Ca^2+^ into the mitochondria and subsequent Ca^2+^ overload when combined with other challenges such as genetic mutations or environmental toxins (Figure 3).

Many genetic causes of PD point to mitochondrial Ca^2+^-overload as a key target in the treatment of PD (Verma et al., 2018; Jung et al., 2020; Lim et al., 2021a; Modesti et al., 2021; Soman and Dagda, 2021). PINK1, which is encoded by a gene whose mutation causes autosomal recessive PD, is a mitochondrially targeted protein that was shown to regulate Ca^2+^ efflux from the mitochondria (Gandhi et al., 2009). Genetic knockdown or knockout of PINK1 resulted in impairment of Ca^2+^ extrusion from the mitochondria in neurons, coupled with increases in ROS production (Gandhi et al., 2009) (Figure 3). Mitochondria purified from PINK1 knockout mice had reduced Ca^2+^ loading capacities and enhanced mPTP activation (Akundi et al., 2011). PINK1 KO midbrain neurons are susceptible to excitotoxic dopamine treatment, but were shown to be protected by NCLX overexpression *in vitro* (Kostic et al., 2015). Deletion of MCU in zebrafish prevented loss of DA neurons in the PINK1 and MPTP models of PD, supporting the potential of targeting MCU for the prevention of PD progression (Soman et al., 2017; Soman et al., 2019). Further support of the role of MCU and mitochondrial Ca^2+^ regulation in PD comes from recent work reporting that MICU1 is regulated by the E3 ubiquitin ligase Parkin, whose mutation leads to another form of familial PD (Matteucci et al., 2018). Overexpression of Parkin reduces MICU1 expression and leads to reduced MICU2 expression due to its dependence on forming dimers with MICU1. Given the critical role of Parkin in regulating MICU1 expression, its mutations could lead to disrupted assembly of the MCU complex and abnormal handling of mitochondrial Ca^2+^ (Matteucci et al., 2018) (Figure 3).

The most common familial form of PD is caused by mutations in leucine-rich repeat kinase 2 (LRRK2) (Lee et al., 2019; Kovacs et al., 2021). Experiments in astrocytes identified that mutant LRRK2 modulates sarco/endoplasmic reticulum Ca^2+^ ATPase (SERCA) function and reduces ER Ca^2+^ uptake (Lee et al., 2019). The reduced uptake of Ca^2+^ into the ER increases ER stress and leads to mitochondrial Ca^2+^ overload (Figure 3) (Lee et al., 2019; Kovacs et al., 2021; Modesti et al., 2021). Human fibroblasts from patients with LRRK2 mutations show upregulation of MCU and MICU1, and mitochondria have increased Ca^2+^ uptake (Verma et al., 2017; Modesti et al., 2021). In SH-SY5Y cells with mutant LRRK2, targeting MCU with inhibitors or genetic knockdown reduced Ca^2+^ uptake and improved neurite growth and stability (Verma et al., 2017). These studies suggest that targeting mitochondrial Ca^2+^ uptake or release may be beneficial in delaying the onset or slowing the progression of PD in humans.

The above-described experiments show the importance of Ca^2+^ regulation and the impact that its dysfunction can have in the progression of PD (Figure 3). Although targeting mechanisms of Ca^2+^ regulation to inhibit PD progression holds promise, additional work is needed to determine how effective this would be in preventing the PD progression. The Ca_v_1.3 inhibitor isradipine has shown to prevent the onset and progression of PD in rodent models of PD (Chan et al., 2009), but has failed to delay clinical progression and onset in clinical trials (2020b; Venuto et al., 2021). Studies have suggested that certain L-type Ca^2+^-channel inhibitors used in patients with hypertension to some extent reduce the risk of PD, but currently no FDA approved drugs available to selectively target Ca_v_1.3 in humans (Chan et al., 2009). Another way of preventing Ca^2+^ overload and excitotoxicity is by reducing NMDA receptor activity. The drug amantadine is a NMDA receptor antagonist that is approved for the treatment of PD induced dyskinesia (Schwab et al., 1969; Olanow et al., 2009). While the clinical use of amantadine is to help reducing symptoms of PD, it could aid in delaying disease progression due to its potential effect on reducing excitotoxic Ca^2+^ signaling through NMDA receptors (Olanow et al., 2009). It is also possible that targeting the MCU complex would be useful in the prevention of PD progression, the approach that showed efficacy in zebrafish models of PD (Soman et al., 2017; Soman et al., 2019). Drug targets of MCU and their efficacy in PD is also discussed in recent reviews (Dey et al., 2020; Woods and Wilson, 2020). However, further testing of these hypotheses in mouse models of PD and humans will require more selective inhibitors of Ca^2+^ regulating proteins.

### Mitochondrial Ca^2+^ regulation in amyotrophic lateral sclerosis

Amyotrophic lateral sclerosis (ALS) is a fatal neurodegenerative disorder that results in progressive loss of motor neurons throughout the brain and spinal cord, leading to death typically within 2.5–3 years after its initial diagnosis (Rosen et al., 1993; Bezprozvanny, 2009; Fu et al., 2018). Although most cases of ALS occur sporadically, approximately 5–10% cases of ALS are familial, with mutations in the Cu/Zn superoxide dismutase (SOD1) gene accounting for approximately 20% of familial ALS cases (Rosen et al., 1993; Cook and Petrucelli, 2019). These mutations lead to toxic accumulation of SOD1 on the mitochondria leading to cell death (Van Den Bosch et al., 2006; Bezprozvanny, 2009). Notably, in mice and rats mutations in SOD1 lead to motor neuron disease similar to familial ALS (Gurney et al., 1994; Howland et al., 2002). Distinct populations of motor neurons are vulnerable in ALS. The neurons most affected are fast-fatigable motor neurons; the most resistant are slow motor neurons, and distinct brainstem motor nuclei populations also remain mostly unaffected (Fu et al., 2018). These findings suggest that, as is the case for PD, a unique neuronal physiology may lead to vulnerability to Ca^2+^ overload in ALS. Spinal motor neurons express lower levels of the Ca^2+^ binding proteins calbindin D_28K_ and parvalbumin (Shaw and Eggett, 2000; Appel et al., 2001). Consistent with this notion, the motor neurons of the brainstem nuclei that are spared from ALS degeneration have higher levels of Ca^2+^ binding proteins (Van Den Bosch et al., 2006; Bezprozvanny, 2009). Further support for the importance of Ca^2+^ binding proteins in motor neurons is that the overexpression of rat parvalbumin in SOD1 mutant mice both delayed the onset of symptoms and prolonged survival of a mouse model of familial ALS (Beers et al., 2001). Increasing the expression of calbindin D_28K_, with glia derived neurotrophic factors, protected motor neurons of spinal cord slices from glutamate induced toxicity (Spruill and Kuncl, 2015).

In addition to the differences in the expression of Ca^2+^-binding proteins, motor neurons have been shown to contain glutamate AMPA receptors that are more permeable to Ca^2+^, thereby adding to their susceptibility to excitotoxicity (Van Den Bosch et al., 2006). This was found to be due to a reduction in the number of GluA2 receptor subunits present within the motor neurons (Shaw and Eggett, 2000; Van Den Bosch et al., 2006). The level of GluA2 subunit expression has been shown to alter lifespan in SOD1 ALS mice. Crossing GluA2 knockout mice with SOD1 mice shortens their lifespan and overexpressing GluA2 increases their lifespan (Van Den Bosch et al., 2006). Along with decreases in the expression of the GluA2 subunits, iPSC derived motor neurons containing the familial *C9orf72* mutation showed increased expression of GluA1 subunits, further contributing to a reduced GluA2/GluA1 ratio and increased Ca^2+^ permeability of AMPA receptors (Selvaraj et al., 2018). A recent assessment of post-mortem tissue from ALS patients found that AMPA receptor subunits were disrupted in both familial and sporadic ALS cases (Gregory et al., 2020). Familial *C9orf72* mutations and sporadic cases showed increased GluA1 expression, whereas SOD1 mutation cases showed reduced GluA2 expression (Gregory et al., 2020). While there appear to be differences in the AMPA receptor subunits expressed in ALS patients, the common denominator is a reduced GluA2/GluA1 ratio that leads to enhanced Ca^2+^ permeability of the receptors (Figure 3).

The excessive Ca^2+^ influx associated with ALS combined with lower expression of Ca^2+^-binding proteins in motor neurons, leave mitochondria in these neurons at high risk of Ca^2+^ overload (Figure 3). Mitochondria Ca^2+^ buffering is dysfunctional in SOD1 mutant mice (Damiano et al., 2006). In mitochondria of the brain and spinal cord, dysfunction in the form of decreased Ca^2+^ loading capacity occurs much earlier than the onset of symptoms and significant motor neuron loss (Damiano et al., 2006). This was supported by a subsequent study that found that mitochondria of motor neurons of pre-symptomatic SOD1 mutant mice became more depolarized from Ca^2+^ entry in response to repeated stimulations (Nguyen et al., 2009). Using motor neurons derived from induced pluripotent stem cells of patients with two different ALS causing mutations (*C9orf72* and *TARDBP*), it was determined that the neurons had enhanced Ca^2+^ release following stimulation and both mutations also led to upregulation of Ca^2+^ permeable AMPA and NMDA receptors (Dafinca et al., 2020). Interestingly, both mutations resulted in higher levels of MICU1 compared to MICU2, with *C9orf72* mutations reducing MCU and MICU2 expression, whereas *TARDBP* increasing MICU1 expression, altogether resulting in reduced uptake of Ca^2+^ into the mitochondria (Dafinca et al., 2020). In hypoglossal motor neurons, mitochondrial Ca^2+^ uptake is impaired later in disease progression, and cytosolic Ca^2+^ clearance is enhanced relative to that in neurons resistant to ALS pathology (Fuchs et al., 2013). An additional analysis showed that in late stage SOD1 mutant mouse hypoglossal motor neurons, not only are MCU and MICU1 upregulated, but so are Letm1, UCP2, and NCX1, one of the plasma membrane Na^+^/Ca^2+^ exchangers (Mühling et al., 2014). As the disease progresses motor neurons attempt to compensate for increased cytosolic Ca^2+^ concentrations by upregulating mechanisms to improve mitochondrial buffering, but also extrusion across the plasma membrane. However, excessive mitochondrial Ca^2+^ load can lead to activation of the mPTP. Notably, in SOD1 mutant mice the mPTP-promoting protein cyclophilin D is upregulated, the mitochondria are swollen, and mPTP can be activated. Deleting a single copy of cyclophilin D gene in SOD1 mutant mice led to delayed disease onset and prolonged lifespan when compared SOD1 mutant mice alone (Martin et al., 2009). These latter findings suggest that inhibiting mitochondrial Ca^2+^ uptake and mPTP activation may be beneficial in preventing the onset and progression of ALS.

The challenge with ALS is that many potential treatments have failed in clinical trials. For example, the FDA approved drug Riluzole has led to only a modest increase in lifespan (about 3 months) (Bezprozvanny, 2009; Smith et al., 2019). The mechanism of action of this drug is not completely clear, but it appears to inhibit glutamate NMDA receptors, an effect that can reduce the overall influx of Ca^2+^ into the cell (Bezprozvanny, 2009; Smith et al., 2019). Many other approaches targeting mitochondrial function and ROS production have failed in clinical trials (Smith et al., 2019). The importance of Ca^2+^ overload in ALS has been extensively discussed, and while studies have shown changes in MCU/MICU1/MICU2 expression in ALS (Fuchs et al., 2013; Mühling et al., 2014; Dafinca et al., 2020), studies targeting mitochondrial Ca^2+^ uptake and release mechanisms have been limited (Tadić et al., 2019). One of the only studies targeting MCU in SOD1 mutant mouse neurons showed that MCU expression is higher in cultured SOD1 mutant motor neurons vs. control motor neurons, but MCU is downregulated in adulthood (Tadić et al., 2019). The application of KN-62, a selective inhibitor of the Ca^2+^/calmodulin-dependent protein kinase II (CaMKII), protected against kainic acid-induced excitotoxity *in vitro*, and this appeared to involve a reduction in MCU expression (Tadić et al., 2019). Although this study holds promise for targeting the MCU complex in ALS, further studies are needed to determine its potential as a therapeutic target.

### Summary and perspectives

Mitochondrial Ca^2+^ buffering is essential for normal neuronal function and dysfunctional Ca^2+^ buffering and subsequent Ca^2+^ overload is common to many neurological diseases. Thanks to the recent identification of the components of the MCU complex, targeting the mitochondrial Ca^2+^ uptake and release machinery has become possible and has helped to refine the roles of these processes in neuronal function, as well as enable investigation of the effectiveness of targeting these proteins in disease prevention. Despite progress, many major questions remain unanswered in defining the roles of specific components of mitochondrial Ca^2+^ uptake and release systems in neurodegenerative disorders, epilepsy, stroke and other neurological conditions. Recent advancements in genetic targeting of all the major components of mitochondrial Ca^2+^ transport have provided important tools for addressing these questions. Thus, an important task for future research would be to systematically examine the impact of deletion or overexpression of MCU, MCUb, MICU3 and other components of mitochondrial Ca^2+^ transport in animal models of AD, PD, ALS, epilepsy, and other neurological conditions.

Even though this review focused primarily on neurons, it is important to point out that glial cells, such as astrocytes and microglia, also play crucial roles in epilepsy and neurodegenerative diseases as highlighted in many excellent reviews on this topic (Salter and Stevens, 2017; Patel et al., 2019; Santello et al., 2019; Escartin et al., 2021). Equally important is that Ca^2+^ signaling and mitochondria Ca^2+^ transport play crucial roles in these cells in the context of many neurological diseases (Shigetomi et al., 2019; Lim et al., 2021b; Heuser and Enger, 2021; Sobolczyk and Boczek, 2022). Thus, another task for future studies would be to define cell-type specific roles of MCU and other molecular components of mitochondrial Ca^2+^ transport in neurological diseases by genetically targeting these molecules in neurons, glia, and immune cells.

Another important line of future research would be development of potent and selective pharmacological tools that target MCU. Although a commonly used MCU inhibitor Ru360 is both potent and selective in *in vitro* assays, its poor plasma membrane permeability and low stability in aqueous solutions limit its applicability for *in vivo* research (Hajnoczky et al., 2006; Woods and Wilson, 2020; Marta et al., 2021). A recently developed analog of Ru360, Ru265, shows improved potency and much more readily penetrates through plasma membrane (Woods and Wilson, 2020). The latter property is mediated by a ubiquitously expressed organic cation transporter 3 (OCT3) (Woods et al., 2020). Notably, administration of Ru265 provided a neuroprotective effect *in vivo* in mice subjected to hypoxic/ischemic brain injury (Novorolsky et al., 2020). Currently, only a few of the available pharmacological inhibitors of MCU are translatable to human medicine. One FDA-approved drug has been shown to inhibit MCU is mitoxantrone (Arduino et al., 2017), but it is also an inhibitor of topoisomerase II and chemotherapeutic with high cytotoxicity (Woods and Wilson, 2020). Mitoxantrone is approved as a treatment for relapsing-remitting multiple sclerosis but may carry some risk for cardiotoxicity as well as risk for the development of cancers (Scott and Figgitt, 2004; Buttmann et al., 2016; Chartier et al., 2018). Interestingly, mitoxantrone improved neurological symptoms and delayed the progression of multiple sclerosis in patients while also being reasonably well tolerated (Scott and Figgitt, 2004). Given its ability to inhibit MCU, it could be an interesting candidate to test in ALS due to its similarities with multiple sclerosis. Another recent screening of FDA-approved compounds has identified two drugs that modulate MCU activity: amorolfine, which promotes mitochondrial uptake of Ca^2+^, and benzethonium, which inhibits this process (De Mario et al., 2021). Since mitochondrial Ca^2+^ overload is a common neurotoxic mechanism implicated AD, PD, ALS, and epilepsy (Figure 3), it will be critical for future experiments to test the efficacy of mitoxantrone, amorolfine, and benzethonium in animal models of these neurological diseases. The major bottleneck in investigating the efficacy of targeting Ca^2+^ uptake in human disease is that only a few drugs and small-molecule inhibitors of the uptake and release mechanisms are currently available. Thus, there is a critical need to screen for such agents, and to develop small-molecule inhibitors that could potentially be effective in treating neurologic diseases in which Ca^2+^ overload is a key problem.
